# Face Context Advantage Explained by Vernier and Separation Discrimination Acuity

**DOI:** 10.3389/fpsyg.2012.00617

**Published:** 2013-01-21

**Authors:** Michael Vesker, Hugh R. Wilson

**Affiliations:** ^1^Department of Biology, Centre for Vision Research, York UniversityToronto, ON, Canada

**Keywords:** face context advantage, vernier acuity, separation discrimination, holistic face processing, face geometry

## Abstract

Seeing facial features in the context of a full face is known to provide an advantage for perception. Using an interocular separation perception task we confirmed that seeing eyes within the context of a face improves discrimination in synthetic faces. We also show that this improvement of the face context can be explained using the presence of individual components of the face such as the nose mouth, or head-outline. We demonstrate that improvements due to the presence of the nose, and head-outline can be explained in terms of two-point separation measurements, obeying Weber’s law as established in the literature. We also demonstrate that performance improvements due to the presence of the mouth can be explained in terms of Vernier acuity judgments between eye positions and the corners of the mouth. Overall, our study shows that the improvements in perception of facial features due to the face context effect can be traced to well understood basic visual measurements that may play a very general role in perceptual measurements of distance. Deficiencies in these measurements may also play a role in prosopagnosia. Additionally, we show interference of the eyebrows with the face-inversion effect for interocular discrimination.

## Introduction

As most people are able to accurately perceive and recognize numerous faces with a high degree of accuracy, the mechanism by which we perceive and categorize information relating to the features of the face has been the subject of intense study. One dominant theory that has emerged is that faces may be perceived holistically as a single “gestalt” rather than perceiving the information describing the various features of the face separately (Tanaka and Farah, 1993). This perception of the whole as opposed to a collection of parts has been demonstrated in experiments showing that people were much more accurate at recognizing parts of the face in recognition tasks when presented with a complete face as opposed to a jumbled face with the parts in differing configurations, or with some parts absent (Tanaka and Farah, 1993; Leder and Carbon, 2004). In addition, complete vertical inversion of the face has also been shown to be detrimental to face perception (Leder et al., 2001).

These findings raise the question: just what is necessary to achieve holistic perception of the face? One study (Kemp et al., 1990) has shown that removing the outline of the head destroys the inversion effect in an interocular distance perception task. This indicates that head-shape is important for accurate perception of interocular distance, leading us to select interocular distance discrimination as the focus in our study. Additionally, a number of other studies have shown that perception of the eye region is impaired in a number of prosopagnosic patients (Caldara et al., 2005; Bukach et al., 2008; Rossion et al., 2009). It has even been proposed that sufferers of prosopagnosia are unable to accurately perceive configural information as a result of an impairment of the mechanisms of holistic perception (Ramon and Rossion, 2010). Finally, curvature discrimination is reportedly compromised in prosopagnosia (Kosslyn et al., 1995).

For simplicity we decided to focus primarily on one aspect of holistic perception: the importance of perceiving the test stimulus in the context of a face for extracting configural information. In our experiments we utilized synthetic faces (Wilson et al., 2002) which gave us more control over our stimuli than photographs. We chose to work with simple circles bandpass filtered by a difference of Gaussians (DOGs, see Eq. 1) as opposed to the more natural looking eyes of the synthetic faces, so that we could contrast how they are perceived independently of face context compared to the full face context. Our first experiment verified that the DOGs we used to replace the synthetic eyes functioned similarly to eyes within a face. We accomplished this by comparing separation discrimination between the DOGs and the synthetic eyes alone. We also compared interocular distance discrimination acuity between eyes and DOGs in the context of a full face, and obtained similar results.

Having verified that the DOGs could act as a viable substitute for the eyes in the context of a face, we ran additional experiments using the DOGs within the synthetic face. This time we compared a number of conditions with various parts of the face systematically removed. We compared the performance levels across these conditions to determine what impact the removal of these features had relative to the full face context, as well as to the DOGs alone. We found that when shown together with either the mouth, nose, or head-outline, the DOGs showed similar performance levels to the full face. Furthermore, when examined in terms of the mean separation from the DOGs to the closest horizontal features in the nose and head-outline cases, the thresholds agreed with threshold levels predicted by Weber’s law from previous studies (Westheimer and McKee, 1977; Watt and Morgan, 1983; Westheimer, 1984). We also found that when viewed with the mouth the DOGs showed thresholds that can be explained by Vernier alignment-judgments between the DOGs and the corners of the mouth in accordance with previous work by Beck and Halloran (1985). These findings lead us to suggest that the face context advantage in interocular perception is not a result of the presence or absence of particular elements of the face. Instead, it depends the availability of reference points, similar to the perception of separation of simple objects.

We also conducted inversion tests to determine what importance the face context may play for inversion effects in our interocular perception task. We found that the presence of eyebrows may degrade performance in inverted faces by slowing the perception of the eye region.

## Materials and Methods

All stimuli were viewed on a 17′′ Macintosh LCD monitor (maximum 75 Hz refresh rate) at a viewing distance of 131 cm. At this viewing distance and a resolution of 1024 × 768, each pixel subtended 1.15 arcmin. Mean screen luminance was 84.45 cd/m^2^.

**Figure 1:**
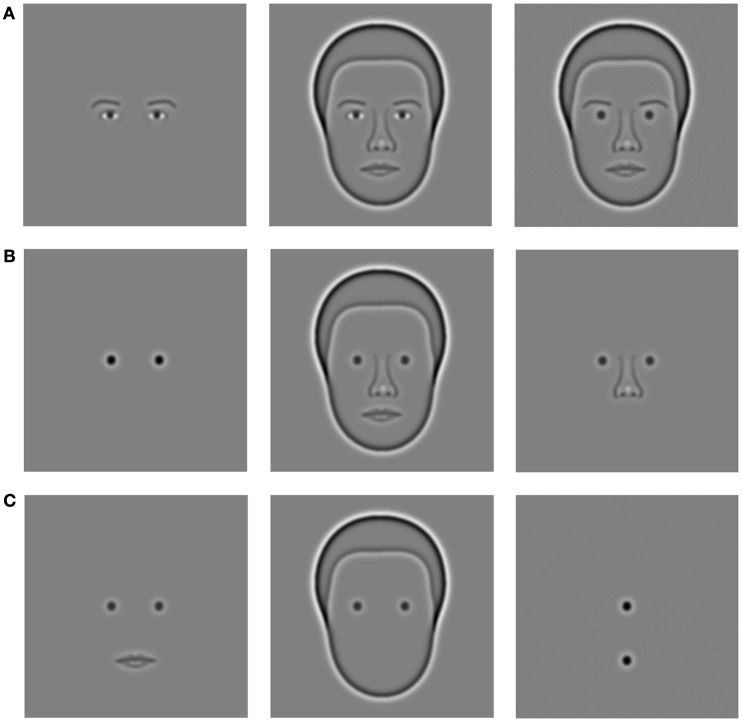
**Major stimuli used for experiments**. Row **(A)** (left to right): eyes alone, face with eyes, face with DOGs, and eyebrows. Row **(B)** (left to right): DOGs alone, face with DOGs, nose and DOGs. Row **(C)** (left to right): mouth and DOGs, outline and DOGs, vertical DOGs.

All face stimuli used in our experiments were synthetic faces (Wilson et al., 2002) originally created by taking 37 measurements of facial features from a database of photographs (see Figure 1). The base face template we used was constructed from the mean dimensions of the male faces in the database. The resulting face was bandpass filtered using the same method as described by Wilson et al. to give us the final images shown to the subjects. The radially symmetric filter we used had a 2 octave bandwidth and a peak frequency of 10 cycles per mean face width (Wilson et al., 2002). These values have been shown to be optimal in previous studies of face recognition (Gold et al., 1999; Näsänen, 1999). The filter is described by a concentric difference of Gaussians with a sigma value of 0.104° and filter radius *R*:

(1) $$DOG\left(R\right)=1.26e^{\frac{-R^{2}}{\sigma^{2}}}-0.26e^{\frac{-R^{2}}{{\left(2.2\sigma \right)}^{2}}}$$

At our viewing distance of 131 cm face width at eye level was 4.7°, giving us 2.1 cycles per degree.

**Figure 2:**
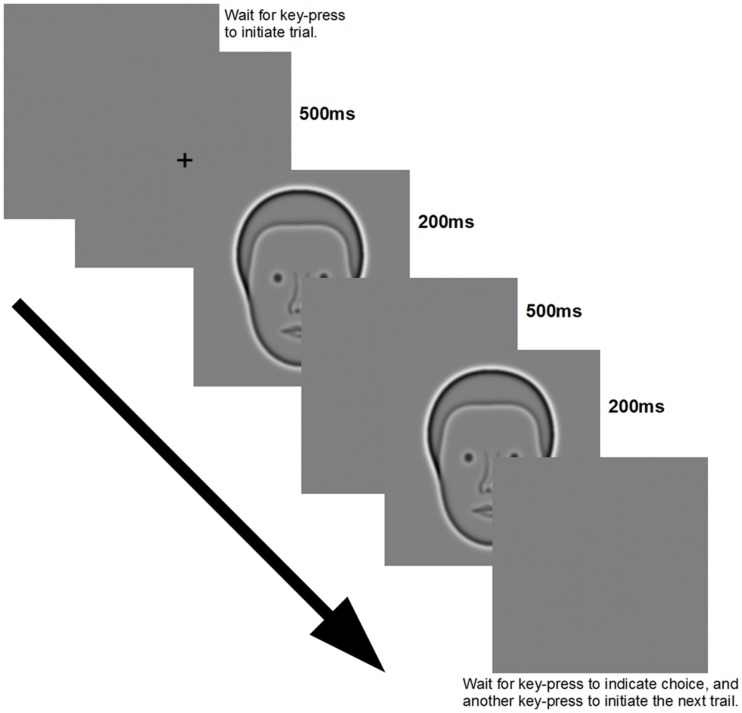
**Presentation sequence and image display duration used for all experiments**.

Except when specified otherwise, all experiments consisted of multiple subject-initiated (via key-press) trials. Upon the initiation of each trial the subject was shown a fixation cross (500 ms), followed by two presentations of stimuli (200 ms each) separated by a blank screen at mean luminance (500 ms), followed by another blank screen (see Figure 2). The subject then entered a key-press to indicate which of the two stimuli had a wider separation between the objects being discriminated (either eyes or DOGs). After entering their choice, the subject pressed a key to initiate the next trial. One of the two presented stimuli always had the tested objects at a fixed reference distance of 125 arcmin, while the other had the objects at one of five increments or decrements relative to the reference distance. In total each increment was tested in 40 trials for each condition (200 trials total per condition) in a single block. Response data from increments and decrements of the same magnitude were combined into a single measurement. The order of presentation was always random. All results were analyzed by repeated measures ANOVA and Fisher’s LSD *post hoc* test.

**Figure 3:**
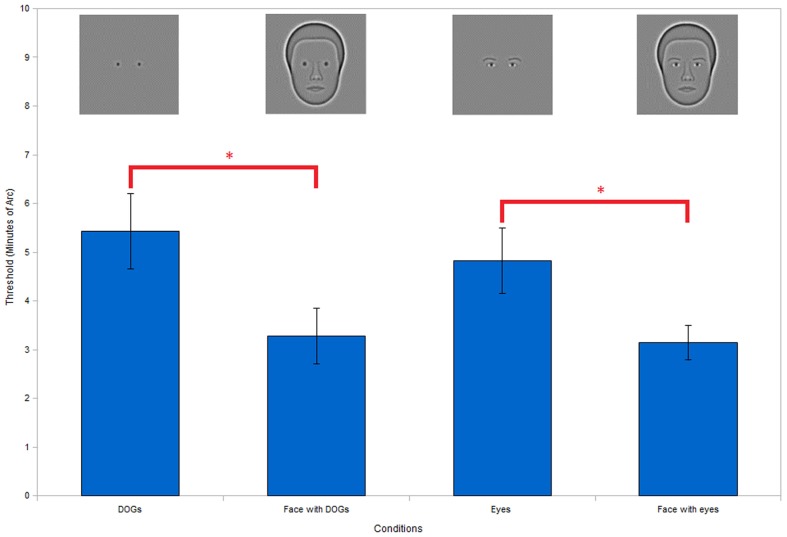
**Comparing the differences between DOGs and eyes, and assessing face context effect**. No significant difference was found between DOGs and eyes or face with DOGs and face with eyes, indicating equivalence between DOGs and eyes. Both DOGs and eyes show significantly better performance within faces than in isolation, thus demonstrating clear face context effects. (**P < 0.01; *P < 0.05).

**Figure 4:**
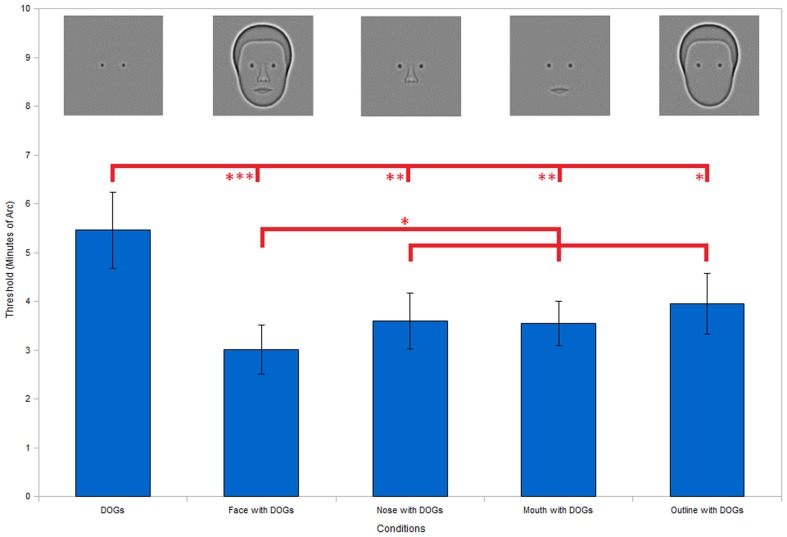
**Incomplete facial stimuli thresholds**. Results show significantly higher discrimination thresholds for the DOGs alone condition compared to the full face condition, as well as all three conditions with only a single facial feature (nose, mouth, or outline) present in addition to the DOGs. None of the three single feature conditions show significant differences from the face condition individually. However, collecting the data from all three condition into a single set shows a significantly higher threshold than the face condition. Error bars represent standard error. (***P < 0.001; **P < 0.01; *P < 0.05).

**Figure 5:**
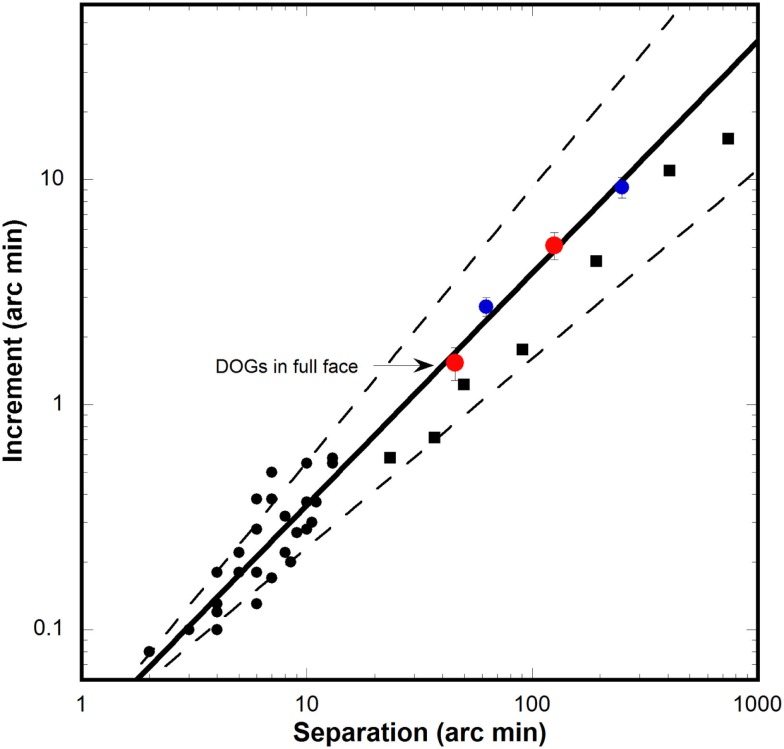
**Separation discrimination data from current and past studies**. Black circles: discrimination increment thresholds for horizontal separation of two vertical bars from three studies (Westheimer and McKee, 1977; Watt and Morgan, 1983; Westheimer, 1984). Solid line: power function fitted to vertical bar data (follows Weber’s law). Dashed line: 95% confidence intervals for power function. Red circles: threshold for DOGs alone at 125 arcmin (upper circle) and threshold from full face condition at mean horizontal distance to nose (45.4 arcmin). Blue circles: threshold for DOGs alone at 62.5 and 250 arcmin. Black squares: data for discrimination of vertical separation of two horizontal bars (Burbeck, 1987).

**Figure 6:**
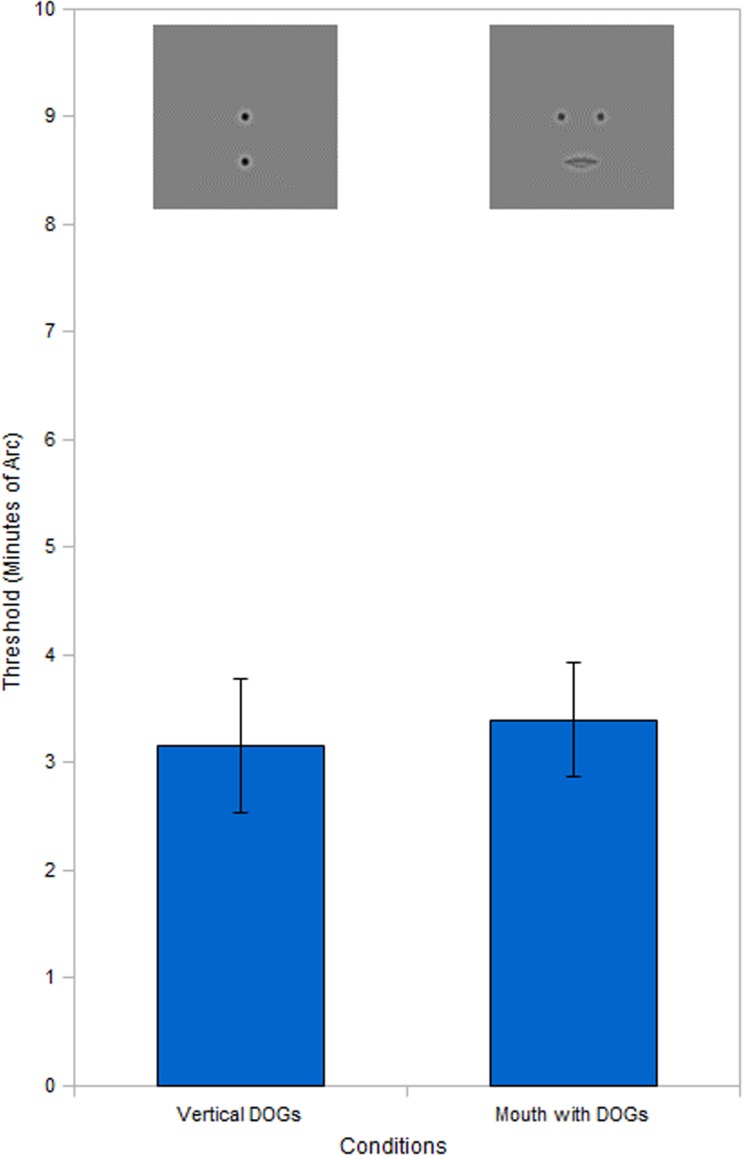
**Vernier acuity comparison**. Results show no significant difference between the mouth with DOGs condition and the vertical DOGs. Threshold data for vertical DOGs condition obtained by using probability summation on the raw threshold data. Error bars represent standard error.

**Figure 7:**
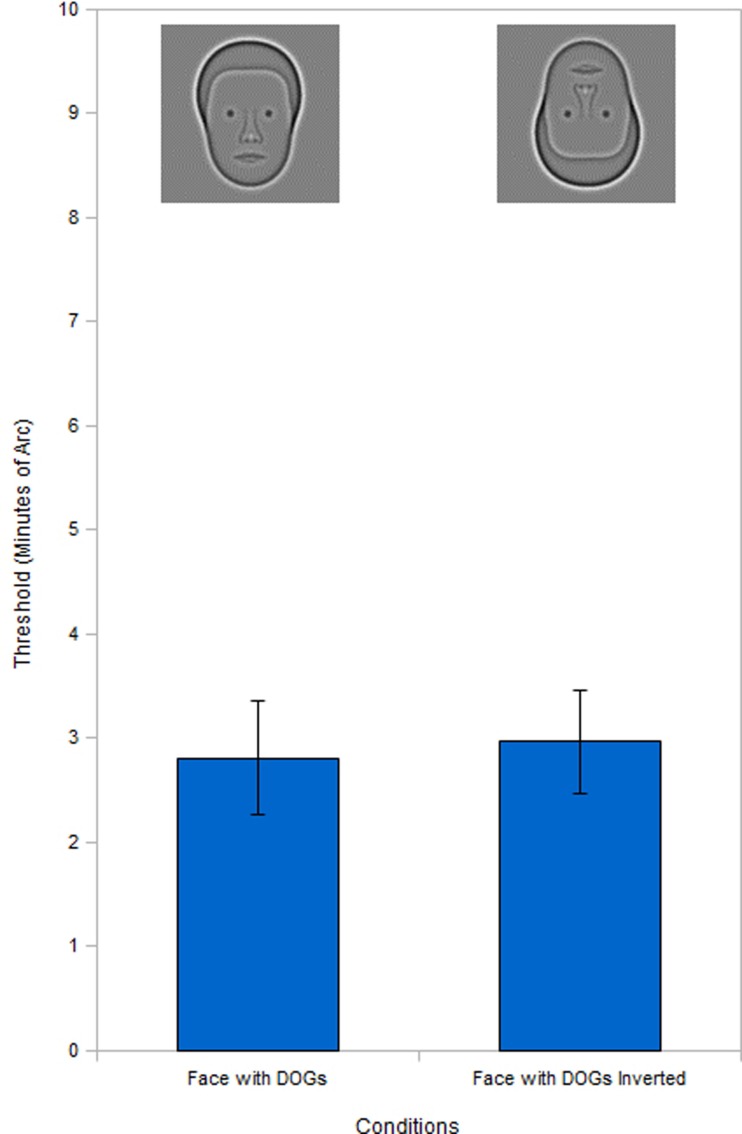
**Full face inversion**. Inversion test of the face with DOGs condition shows no significant inversion effect.

**Figure 8:**
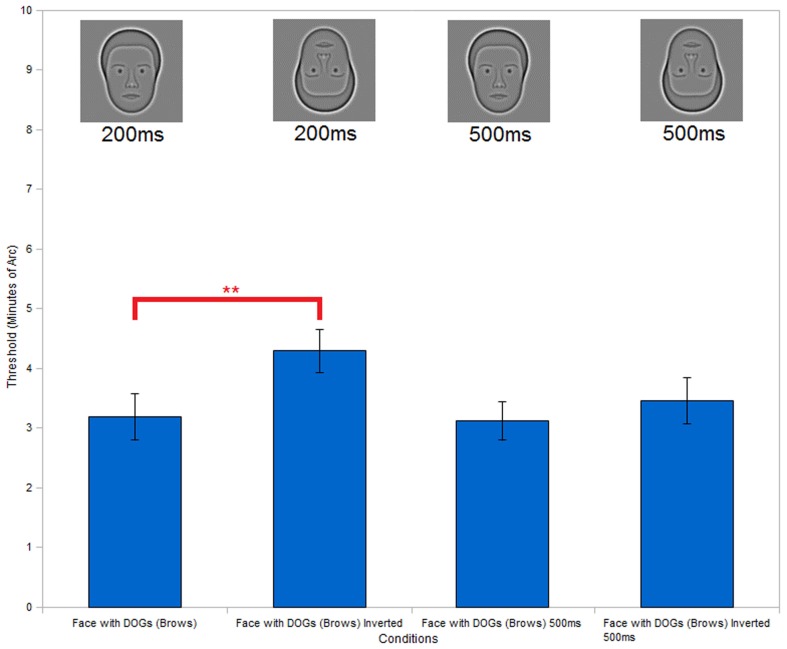
**Full face with eyebrows inversion**. Inversion test of the face with DOGs condition (with added eyebrows) shows a significant improvement in performance of the upright condition versus the inverted condition at an exposure duration of 200 ms. Repetition of the inversion test with the same stimuli and increased exposure duration (500 ms) shows no significant differences between the upright and inverted conditions. (**P < 0.01).

Participants in this study consisted of eight subjects: three females and five males between the ages of 22 and 36 (mean age 27). The data presented in Figure 3 were collected from all subjects. The data presented in Figure 4 do not include the thresholds from one of the male subjects as he was unable to complete all the experimental conditions. All newly collected data presented in Figures 5 and 6 were collected from three male and two female subjects. All data presented in Figures 7 and 8 were collected from three male and three female subjects.

## Results

In order to better examine the face context effect we simplified the eyes to improve control over our stimuli. To do this we replaced the natural synthetic eyes with DOGs (Eq. 1). To verify that the DOGs were being perceived as the eyes in the context of the face we carried out an experiment in which we compared our subjects’ thresholds across four conditions: eyes alone, face with eyes, DOGs alone, and face with DOGs. This comparison allowed us to simultaneously assess the presence of the face context effect for both the eyes and DOGs, by comparing each of those conditions against their respective face conditions.

ANOVA of the results (Figure 3) showed a significant effect of condition [*F*(3, 18) = 6.54, *P* < 0.01]. *Post hoc* comparison of the conditions with DOGs in isolation against the eyes in isolation showed no significant differences (*t*_18_ = 0.96, *P* = 0.35). Comparing the conditions of the face with DOGs versus the face with eyes also showed no significant differences (*t*_18_ = 0.22, *P* = 0.83).

When comparing the thresholds of the eyes in isolation versus the face with eyes, we found significantly better performance in the face condition (*t*_18_ = 2.68, *P* < 0.05). Similarly, we also found a significant improvement in performance in the face with DOGs versus the DOGs in isolation (*t*_18_ = 3.43, *P* < 0.01).

Seeing no significant differences between the DOGs and eyes in both the isolated and face conditions allowed us to conclude that DOGs can function as eyes in the context of a face. This demonstrated that our use of DOGs in place of eyes within the face is a valid substitution for the experiments to follow.

The comparisons between the eyes and the face with eyes, as well as between the DOGs and face with DOGs both showed clear performance advantages in the case of the face conditions. This demonstrates a clear face context effect for both the eyes and DOGs, which we investigate further in the following experiments.

Having established equivalency between the eyes and circles in the context of the face, we used DOGs in subsequent studies. First, we removed facial features to determine what importance each individual feature had for the face context effect. In each case of feature removal we left only a single feature in place to be observed along with the DOGs. Five conditions were tested: isolated DOGs, full face with DOGs, nose with DOGs, mouth with DOGs, and head-outline with DOGs.

As in the previous experiment, ANOVA of the results (Figure 4) showed a significant effect of condition [*F*(4, 20) = 4.39, *P* < 0.05). The condition of the face with DOGs showed significantly (*t*_20_ = 3.91, *P* < 0.001) better performance versus the DOGs alone (Figure 4) in the *post hoc* analysis. Likewise, the nose, mouth, and head-outline single feature conditions all showed significantly lower thresholds compared to the DOGs alone: *t*_20_ = 2.97, *P* < 0.01, *t*_20_ = 3.06, *P* < 0.01 and *t*_20_ = 2.40, *P* < 0.05, respectively (Figure 4). However, none of the three single feature conditions individually showed higher thresholds than the whole face condition. This indicated that each of the three individual features contributed to the face context effect.

In a further analysis, data from the nose, mouth, and outline conditions were grouped and compared with the whole face condition. When combined into a single data group in this manner, the single feature conditions did show significantly worse performance (*t*_17_ = 2.43, *P* < 0.05) than the face condition (Figure 4). This suggests that while individual features may be capable of producing a face context effect in isolation, the magnitude of the effect increases when multiple features are combined through probability summation, a point to which we return in the discussion.

Having observed that the three conditions with single features each produced a face context effect, we hypothesized that the threshold levels were primarily dependent upon the presence of useful reference points. Of the three individual features, we hypothesized that the nose and head-outline were improving interocular distance discrimination by providing reference points that lie on the same horizontal line that defines the interocular variation. To test this hypothesis we examined the literature on horizontal separation discrimination of simple objects. Three studies (Westheimer and McKee, 1977; Watt and Morgan, 1983; Westheimer, 1984) examined horizontal separation discrimination of two vertical bars at very small base separations. Since these sets of data were obtained at separations much smaller than the ones in our study, the data were extrapolated to obtain an estimate of the thresholds at our much larger (125 arcmin) base separation. The extrapolation was performed by combining the data from all three studies, and fitting a power function to the data-points. The extrapolation (solid line, Figure 5) had a power law exponent of 1.03, indicating that the data obey Weber’s law. As shown by the upper red symbol our threshold for the separation of two DOGs in isolation nicely fit the extrapolated line, well within the 95% confidence interval of the extrapolation (dashed lines). We found a similarly fit good with the threshold from the full face condition (lower red symbol) when it was plotted at the base separation that corresponded to the mean distance (45.4 arcmin) between each DOG and its nearest parallel feature which was the side of the nose. Because the relevant displacement is between each individual DOG and the nose-edge nearest to it, the threshold was divided by two to reflect the increment for one eye alone.

In order to test the veracity of our extrapolation we also ran an additional experiment using two conditions similar to the isolated DOGs but at different base separation (62.5 and 250 arcmin). The thresholds obtained with these new conditions (blue circles) showed a good fit to our extrapolated trend (Figure 5), indicating that the thresholds measured in our experiments obey Weber’s law for separation discrimination. Additionally, we plotted (Figure 5, black squares) discrimination thresholds from a study (Burbeck, 1987) for vertical separation of two horizontal bars at large base separations and found that they also fit within the 95% confidence interval of our extrapolation.

As seen in Figure 4, the mouth, like the nose and head-outline, also produced a face context effect. This caught our interest because the mouth is positioned below the DOGs and that condition lacks any horizontal reference point that could aid in separation discrimination. This suggests that for the mouth to serve as a useful reference point, a subject would have to judge the change in distance between each DOG and the closest corner of the mouth. In our experimental condition, these points lie 101 min of arc beneath each DOG. We hypothesized that since the DOGs and the ends of the mouth were approximately aligned (vertically) a subject could use a large separation Vernier alignment measure (Beck and Halloran, 1985) on each DOG and the mouth-corner below it.

To test this hypothesis we created a new condition (vertical DOGs, see Figure 1) comprised of two DOGs (like those used in previous experiments) aligned vertically instead of horizontally. The top DOG was positioned horizontally in the center of the screen at the same height as the DOGs in previous experiments. The bottom DOG was positioned directly below the top DOG, at the height corresponding to the corner of the mouth. The experimental setup was the same as previous conditions (two 200 ms exposures separated by 500 ms blank), but with a modified task. The reference exposure in this experiment had the top and bottom DOGs perfectly aligned. The other (misaligned) exposure had the bottom DOG remain in the same position while the top DOG was displaced to the left or to the right in five increments. The subject’s task was to determine which exposure was misaligned.

One additional consideration was that in the condition with the DOGs and mouth the subject could make a judgment using either the left or right mouth-DOG pair in order to make a correct decision. In order to obtain a threshold that would reflect the subject’s performance if two Vernier judgments could be made, we used probability summation (see Eq. 2) on the initial thresholds obtained (mean value = 5.09 arcmin), with *q* representing the shape factor of the psychometric function for each subject (estimated by fitting a Quick/Weibull function to the data), *m* representing the original threshold and *M* representing the final threshold. Range value of *q* was 1.32–1.78 with a mean of 1.49.

(2) $$M={\left[\overset{2}{\underset{i=1}{\sum }}\left(\frac{1}{{\left(m_{i}\right)}^{q}}\right)\right]}^{\frac{-1}{q}}$$

The results (Figure 6) indicate that after probability summation, the vertical DOGs produce a similar threshold to that obtained in the condition with the circles and mouth, with no significant differences found between the two (*t*_4_ = 0.23, *P* = 0.83).

When we tested the full face DOGs condition for the inversion effect we found (Figure 7) that there was no significant difference (*t*_5_ = 0.13, *P* = 0.91) between the upright and inverted conditions. This was unexpected as the inversion effect is well established for faces. One difference between our full face DOGs condition and natural face stimuli was the lack of eyebrows.

To test whether the missing eyebrows were responsible for the lack of inversion effect, we repeated the inversion test with full face DOGs stimuli, but with eyebrows added. With the addition of the eyebrows we found (Figure 8) a significant inversion effect (*t*_5_ = 4.49, *P* < 0.01), prompting us to ask why the presence of eyebrows impeded the discrimination of interocular separation in the inverted condition.

One possible answer is that the addition of eyebrows introduced more information to the eye region. In the upright condition subjects might be able to process the extra information more rapidly due to our expertise with upright faces. In the inverted condition on the other hand, processing the additional information could require additional time, leading to a higher error rate in perception, given the limited exposure times of the stimuli. We tested this hypothesis by repeating the inversion test of the full face DOGs condition (with eyebrows), but with an extended exposure time of 500 ms (compared to the 200 ms exposures used previously). The results (Figure 8) showed no significant inversion effect (*t*_5_ = 0.78, *P* = 0.47) with the longer exposures, thus supporting this explanation.

## Discussion

We fist established the advantage of seeing eyes in the context of a face when discriminating the interocular separation. The results (Figure 3) showed that the face context improved performance by a factor of 1.7. Replacing the eyes with DOGs produced similar performance levels both in the context of a face and in isolation. As a result of these findings, we replaced the eyes with DOGs in order to simplify the stimuli for further examination.

The next experiments were designed to determine which facial features contribute to the face context effect. We created new stimuli featuring DOGs combined with single facial features. By comparing these incomplete face stimuli with the full face as well as the DOGs alone we identified which features contributed to the facial context necessary for the improvement in performance demonstrated in the first experiment. Our results (Figure 4) showed that every one of the features we tested (nose, mouth, and head-outline) was individually capable of providing an improvement over the isolated DOGs (see also Brunas et al., 1990). These findings raised the question of how each individual feature is able to produce the face context effect.

We hypothesized that the nose and head-outline conditions produced face context effects by providing reference points positioned in-line with the displacement of the DOGs. If that is the case, then subjects could use these features to make simple separation discriminations. We tested this hypothesis by comparing our thresholds with data from studies that tested horizontal separation discrimination of two vertical bars (Westheimer and McKee, 1977; Watt and Morgan, 1983; Westheimer, 1984). As the data from these studies were collected at base separations much smaller than the interocular distance used in our experiments, we extrapolated the data from previous studies to our own base separations. Additionally, we plotted data from a study measuring vertical discrimination of horizontal bars (Burbeck, 1987) which fit within the 95% boundaries of our extrapolation. We found that our thresholds for isolated DOGs also fit well within the 95% confidence interval of the extrapolated function (Figure 5). The thresholds from the full face with DOGs condition showed a similarly good fit when plotted as a function of the distance from each DOG to the nearest in-line reference point (side of the nose). We also tested the isolated dogs at two additional separations of 62.5 and 250 arcmin to better establish the trend of data from our experiments. The thresholds from these measurements also showed a strong fit to the extrapolated function. We noted that all data plotted in Figure 5 showed a strong adherence to Weber’s law. These findings supported our hypothesis that the nose and head-outline were being used by subjects as reference points using mechanisms similar to those used in simple measurements such as bar separation.

This left the question of how the condition with the mouth was able to show the face context effect when the mouth lies below the DOGs. We theorized that the performance gain over the isolated DOGs could be explained by the subjects performing a Vernier alignment judgment (Beck and Halloran, 1985) between each DOG and the nearest edge of the mouth. To test this hypothesis we measured the threshold for detecting horizontal displacement of a DOG vertically aligned to a second DOG below it (at a height corresponding to the distance between the DOGs and mouth-corners). However, due to the fact that in the DOGs and mouth conditions each DOG could give the correct response independently, we used probability summation on the thresholds obtained from the vertically aligned circles condition. The resulting calculated thresholds did not show any significant differences when compared to the mouth and DOGs condition (Figure 6), thus supporting our hypothesis.

Overall, these results demonstrate that people can use simple measurements to achieve the face context effect even with incomplete faces so long as some features are present to provide useful reference points. We showed that each of three individual features (head-outline, nose, and mouth) fit within this model and show thresholds similar to that of the full face. When combined into a single data set, the full face does show a significant advantage over each single feature condition (Figure 4). We hypothesize that this advantage of the full face results from probability summation among the three feature conditions. Computations show that probability summation with an exponent *q* = 2 or *q* = 3 in Eq. 2 can account for the threshold of the face with DOGs in Figure 4. This supports the role of probability summation among several features in producing the face context advantage.

Our research also suggests that deficits in perception of the eye region in patients with prosopagnosia (Caldara et al., 2005; Bukach et al., 2008; Rossion et al., 2009) could be linked to deficits in the ability to make simple measurements such as separation of basic shapes and Vernier judgments. This is supported by a study (Barton and Cherkasova, 2005) which showed that five out of six prosopagnosic patients had similar deficiencies in perceiving facial variations (including reduction of the interocular separation) and separation of two dots relative to healthy controls. Another study (Kosslyn et al., 1995) has shown that prosopagnosia can produce deficiencies in the perception of curvature, which can be though of as detection of misalignment similar to Vernier judgments that play a role in the perception of interocular separation. Prosopagnosics might even show larger Weber fractions or other deviations from Weber’s law. This will be the basis of a future study.

It is important to note that the thresholds measured in our experiments not only obey Weber’s law, but also preserve the same value of the Weber fraction as the results from three previous studies on discrimination of horizontal separation (Westheimer and McKee, 1977; Watt and Morgan, 1983; Westheimer, 1984). This is suggestive of the same underlying mechanisms being used for these discriminations. Furthermore, adherence to Weber’s law is consistent with the operation of size constancy for face context effects over a considerable size range.

Ever since it was first reported (Yin, 1969), the inversion effect has been accepted as a property of face stimuli and has been reported for the synthetic faces used in our study (Wilson et al., 2002). It was therefore surprising to discover that the face with DOGs condition did not exhibit this common effect (Figure 7). We initially speculated that this might be due to the removal of the eyebrows in our stimuli. This would agree with previous experiments showing that removal of facial characteristics and substitution of circles in place of eyes destroys the inversion effect (Kemp et al., 1990). We tested the hypothesis that the absence of eyebrows could have caused the lack on inversion effect by conducting an inversion test with eyebrows added to our stimuli. The results (Figure 8) showed that the addition of the eyebrows restored the inversion effect. This finding prompted us to hypothesize that the decrease in performance in the inverted condition with the addition of the eyebrows could be explained in terms of expertise and complexity. The eyebrows add information to the eye region where our subjects focus their attention. In upright faces this would not present a problem, as the extra information is easily integrated holistically thanks to human expertise with upright faces. In inverted faces however, the additional information may not be integrated as efficiently, thus slowing down the whole process. This would have produced a higher error rate in the inverted condition due to the limited exposure duration we used (200 ms). We tested this hypothesis by repeating the inversion test with the eyebrows present but increased the exposure duration of the stimuli to 500 ms. We found (Figure 8) that the increased exposure did eliminate the inversion effect, thus supporting our hypothesis.

Our results are supported by another recent study (Richler et al., 2011) that explored the inversion effect for composite faces. In the original study using composite faces, it was shown that inverted faces fail to exhibit the composite face effect (Young et al., 1987). However, Richler et al. (2011) showed that this failure was a function of exposure duration. Inverted faces exhibited a robust composite face effect very similar to upright faces when the exposure duration was extended significantly. As the composite face effect reflects holistic face processing, both their results and ours suggest that apparent lack of holistic processing for inverted faces results from slower processing that is not fundamentally different in kind from upright face processing.

## Conclusion

Our findings indicate that while the face context as a whole provides a definite advantage for the perception of interocular separation, the mechanism behind this face context advantage can be explained by a combination of basic separation discrimination and Vernier acuity. Individual facial features (head-outline, nose, and mouth in our case) make this possible by providing useful reference points in relation of the eye positions. These points could be used to more accurately determine interocular distance in a manner similar to that used for the perception of distance and alignment between simple shapes completely outside the context of face perception, that have been long established in the literature (Westheimer and McKee, 1977; Watt and Morgan, 1983; Westheimer, 1984; Beck and Halloran, 1985; Burbeck, 1987). We also suggest that deficiencies in the mechanisms of these simple measurements could be the cause of deficiencies in eye region perception in cases of prosopagnosia (Caldara et al., 2005; Bukach et al., 2008; Rossion et al., 2009). Finally, we suggest that inversion effects on interocular separation discrimination are degraded by the presence of eyebrows beneath the eyes. Our data suggest that this may reflect slower processing in the inverted face condition.

## Conflict of Interest Statement

The authors declare that the research was conducted in the absence of any commercial or financial relationships that could be construed as a potential conflict of interest.
